# Constraint-Based Hierarchical Cluster Selection in Automotive Radar Data †

**DOI:** 10.3390/s21103410

**Published:** 2021-05-13

**Authors:** Claudia Malzer, Marcus Baum

**Affiliations:** 1 Data Fusion Group, Institute of Computer Science, University of Göttingen, 37077 Göttingen, Germany; marcus.baum@cs.uni-goettingen.de; 2 Faculty of Engineering and Health, HAWK University of Applied Sciences and Arts Hildesheim/Holzminden/Göttingen, 37085 Göttingen, Germany

**Keywords:** hierarchical clustering, HDBSCAN, constraint-based clustering, semi-supervised clustering, automotive radar

## Abstract

High-resolution automotive radar sensors play an increasing role in detection, classification and tracking of moving objects in traffic scenes. Clustering is frequently used to group detection points in this context. However, this is a particularly challenging task due to variations in number and density of available data points across different scans. Modified versions of the density-based clustering method DBSCAN have mostly been used so far, while hierarchical approaches are rarely considered. In this article, we explore the applicability of HDBSCAN, a hierarchical DBSCAN variant, for clustering radar measurements. To improve results achieved by its unsupervised version, we propose the use of cluster-level constraints based on aggregated background information from cluster candidates. Further, we propose the application of a distance threshold to avoid selection of small clusters at low hierarchy levels. Based on exemplary traffic scenes from nuScenes, a publicly available autonomous driving data set, we test our constraint-based approach along with other methods, including label-based semi-supervised HDBSCAN. Our experiments demonstrate that cluster-level constraints help to adjust HDBSCAN to the given application context and can therefore achieve considerably better results than the unsupervised method. However, the approach requires carefully selected constraint criteria that can be difficult to choose in constantly changing environments.

## 1. Introduction

High-resolution automotive radar sensors are increasingly being used for detection, classification and tracking of moving objects in traffic scenes. Their major benefits are their robustness towards environmental factors such as fog and rain, their wide range and their ability to estimate the speed of an object based on Doppler velocity.

In this context, clustering, i.e., extracting groups of similar data points (which ideally belong to the same object), is particularly challenging. The number of returned radar reflections as well as their density and measured velocity can depend on various factors, such as distance and angle to the sensor, and strongly vary across multiple measurement cycles [1,2]. So far, the density-based algorithm DBSCAN [3] has been the most common choice for extracting dense groups of radar measurements and declaring all others as noise. While HDBSCAN [4], a hierarchical DBSCAN variant and current state of the art in hierarchical clustering, has gained a lot of attention across many different research fields in recent years, it has rarely been used in the context of radar data. While some authors did not consider hierarchical methods because of their high runtime complexity compared to non-hierarchical methods [1,5], Schumann et al. [2] tested HDBSCAN on radar measurements of stationary and moving objects but found it not suitable for their task.

Yet, hierarchical approaches have certain advantages which are worth exploring in the context of radar measurements. Flat clusters can be extracted from hierarchies on very different density levels, while DBSCAN relies on a global distance threshold that ignores levels beyond this value. Moreover, hierarchies provide valuable insight into dependencies between data partitions that can be used in further processing steps. Honer and Schmieder [6] pointed out that the hard decision of a single flat clustering output can decrease performance of multi-target tracking systems [7]. To achieve better results, the authors used a parent-child hierarchy between partition cells of automotive sensor measurements for the implementation of a Gibbs sampler that simultaneously samples from a set of partitions from this hierarchy and the associated mappings.

Against this background, we explore the applicability of HDBSCAN for clustering automotive radar data. In contrast to Schumann et al., we focus on cluster extraction from the hierarchy and integration of background knowledge. To improve results of HDBSCAN’s default unsupervised selection method and for better adjustment to the application context, we introduce a new selection method using cluster-level constraints based on aggregated information from cluster candidates.

We further develop preliminary work from our conference paper [8] by testing this new constraint-based approach in combination with our proposed selection method HDBSCAN($\hat{\epsilon }$), which applies a distance threshold $\hat{\epsilon }$ to the hierarchy. HDBSCAN($\hat{\epsilon }$) can achieve a combination between HDBSCAN clusters, which are independent of this threshold, and $\hat{\epsilon }$-dependent DBSCAN* clusters.

Overall, this article extends the content from our conference paper by multiple aspects, which can be summarized as follows:

- Discussion of HDBSCAN($\hat{\epsilon }$) from the perspective of constraint-based clustering
- Introduction of a cluster-level constraint-based HDBSCAN selection algorithm that can be used with or without the $\hat{\epsilon }$ threshold
- Experiments with automotive radar data from exemplary traffic scenes to explore whether the integration of background knowledge can improve HDBSCAN’s performance in this application context
- Comparison of our cluster-level semi-supervised HDBSCAN version with unsupervised and instance-level semi-supervised HDBSCAN
- Demonstration of different effects when applying $\hat{\epsilon }$ before or after constraint-based cluster selection

After addressing related work in Section 2, we introduce and discuss HDBSCAN($\hat{\epsilon }$) in Section 3, where we also explain general concepts and selection methods of HDBSCAN. In Section 4, we propose our constraint-based HDBSCAN version that is adjusted to clustering automotive radar measurements. We also describe data sets and further methods used in our experiments. In Section 5, we discuss strengths and weaknesses of the tested approaches based on results from our experiments. Section 6 concludes with a summary and perspectives for future research.

## 2. Related Work

As a density-based clustering approach, DBSCAN by Ester et al. [3] can find clusters of arbitrary size and shape, does not require the number of clusters a priori, and classifies points as noise if a given density criterion is not fulfilled. This criterion is defined by two input parameters: the minimum number of neighbors minPts and the distance threshold ϵ (epsilon) that defines the radius of the neighborhood. Points with at least minPts neighbors within the epsilon radius are called core points and form a cluster. Points that are no core points but can be reached from a core point within a distance of epsilon are called border points and also assigned to clusters, while all others are classified as noise.

Kellner et al. [1] proposed a DBSCAN variant for clustering radar data based on a grid with pre-calculated, sensor-specific values for the ratio between radial distance (range) and angular (azimuth) distance. For each grid cell, this ratio is used to determine a local minPts value. The neighborhood radius is either circular as in classic DBSCAN, or adjusted in azimuth direction such that it becomes an ellipsoid that gets narrower and thus contains less points the closer it gets to the sensor.

Schubert et al. [9] used DBSCAN with only minor modifications to cluster radar reflections of moving pedestrians. In a three-dimensional *x*-*y*-*v* space where *x* and *y* refer to Cartesian coordinates and *v* to Doppler velocity, they filtered out stationary reflections below a threshold of 0.1 $\frac{m}{s}$ and applied DBSCAN with minPts=2, ϵ=1 m and an additional velocity threshold of 1 $\frac{m}{s}$.

Wagner et al. [10] proposed another DBSCAN variant called EDBSCAN that derives ellipsoidal footprints from the power of detected objects and merges points with overlapping footprints.

Schumann et al. [2] divided the data space into 30 distance-velocity regions. In each region, they trained the data set by using an adaptive simulated annealing algorithm to optimize parameter values for minPts and three thresholds for distance ($\epsilon_{r}$), velocity ($\epsilon_{v}$), and time ($\epsilon_{t}$). DBSCAN was further modified to prefer moving objects: reflections below a certain velocity threshold were allowed as border points of a cluster, but not as core points. The authors showed that DBSCAN based on learned parameters can outperform DBSCAN with a manually chosen parameter set, as well as unsupervised HDBSCAN with a custom metric in a scaled *x*-*y*-*v*-*t* space.

Li et al. [5] summarized common problems when using global parameters for a DBSCAN clustering window in a three-dimensional range-angle-velocity space: not being able to form a cluster for an object because either the number of reflection points is too low or the distance is too large, having the point cloud for one object split up into separate clusters due to variability in distance or velocity, and having relevant reflections regarded as noise because they are too far away from the cluster. To decrease parameter dependency and improve results, the authors estimated object contours based on clusters from previous clustering cycles and used their dimension and velocity properties to define an additional, adaptive DBSCAN clustering window.

The concept of merging initial clusters in an additional clustering step can also be found in other papers like Schumann et al. [11], where the authors correct initial DBSCAN clusters manually before re-clustering, and in the multi-step radar clustering framework proposed by Scheiner et al. [12]. As first step within this framework, data points with few neighbors and low velocity are filtered out based on manually chosen thresholds. In a *x*-*y*-*v* space and a time window of 0.25 s, the data is then clustered with a modified DBSCAN that adjusts minPts depending on the distance between object and sensor, and only allows reflections to become core points if they exceed a certain velocity threshold as in [2]. A second DBSCAN clustering step with different parameters is used to merge clusters based on properties such as object orientation and estimated velocity.

The current state of the art in hierarchical density-based clustering is HDBSCAN by Campello et al. [4], which constructs a simplified hierarchy of cluster candidates and then selects a set of flat clusters based on local cuts through the tree. Hence, the process of repeating a clustering step to merge initial clusters could be replaced by constructing the hierarchy once with a low minimum cluster size, and then merging certain candidates based on application-specific criteria. We will describe HDBSCAN in more detail in Section 3.

Semi-supervised clustering refers to the integration of background knowledge into an otherwise unsupervised clustering task. Many clustering algorithms have already been extended to run in a semi-supervised way. Typically, background information is expressed in form of instance-level constraints between pairs of data points, which are either of the type must-link or cannot-link [13]. For example, Wagstaff et al. [14] clustered GPS records with the k-means algorithm to detect highway lanes and defined a maximum separation criterion of 4 m as cannot-link constraint to avoid that points from different lanes get merged.

Davidson & Ravi [15] defined the terms ϵ constraint and δ constraint for constraints that enforce a maximum (ϵ) or minimum (δ) distance for points in a cluster. Points that would violate the ϵ constraint are left as singleton clusters. The authors explored the feasibility of satisfying different constraints in agglomerative hierarchical clustering, performed a complexity analysis and proposed the use of a centroid-based distance constraint γ to improve runtime [16,17]. We will include a constraint similar to γ when clustering radar data (Section 4), although not to reduce runtime but rather to avoid merging clusters that are unlikely to represent the same vehicle. In Section 3.2.3, we will also return to the subject of distance constraints in context of our selection method HDBSCAN($\hat{\epsilon }$).

In contrast to traditional instance-level constraints, Campello et al. [18] implemented a semi-supervised HDBSCAN version based on so-called should-link and should-not-link constraints. These are soft rather than hard constraints and used to guide the selection of clusters. Gertrudes et al. [19] further extended this approach by an alternative HDBSCAN selection method that directly uses labels instead of turning them into pairwise constraints. We will explain this version in more detail in Section 3.2.2, and also include it in our experiments. The general difference between using constraints in HDBSCAN and previous hierarchical constraint-based clustering approaches is that HDBSCAN’s density-based hierarchy is not modified and constraints are considered only when extracting flat clusters from the tree.

Cluster-level constraints, which we use in a custom selection approach from the HDBSCAN hierarchy in Section 4, allow to make decisions about merging two clusters based on aggregated information from their instances. Again, previous work about cluster-level constraints in hierarchies (e.g., Davidson & Ravi [16] in context of agglomerative clustering and Ge [20] with a custom tree structure) focused on the construction of constraint-based hierarchies instead of the extraction of clusters from a hierarchy that was built in an unsupervised way. The two mentioned semi-supervised HDBSCAN approaches only focus on instance-level constraints.

## 3. The HDBSCAN Algorithm

HDBSCAN is built on top of a slightly modified version of DBSCAN, called DBSCAN*, which declares border points as noise [4]. Unlike DBSCAN(*), HDBSCAN does not select clusters based on a global epsilon threshold, but creates a hierarchy for all possible epsilon values and thus only requires minPts as single input parameter.

### 3.1. Hierarchy Construction

The constructed HDBSCAN hierarchy is based on mutual reachability distance, which for two points $x_{p},x_{q}$ is

$$max\{d_{core}\left(x_{p}\right),d_{core}\left(x_{q}\right),d\left(x_{p},x_{q}\right)\}$$

where $d(x_{p},x_{q})$ refers to the chosen distance function, e.g., Euclidean distance, and the core distance dcore is defined as the distance of a point to its minPts-nearest neighbor. This approach separates sparse points from others by at least their core distance to make the clustering more robust to noise.

The data set can be represented as a graph with data points as vertices, connected by edges with their mutual reachability distances as weights. A hierarchical tree structure is obtained by creating a minimum spanning tree and sorting its edges by mutual reachability distance. HDBSCAN then constructs a simplified version of this hierarchy, called condensed cluster tree. Starting from the root, each cluster split is regarded as a true split only if both nested child clusters contain at least minPts points. If they are both smaller than minPts, the cluster is considered as having disappeared at this density level. If only one child has less than minPts data points, these instances are classified as noise while the larger child is considered as still being part of its parent. This simplification process results in a hierarchy of DBSCAN* candidate clusters at different density levels, where the leaf nodes are the candidates with the lowest epsilon values that cannot be split up any further with respect to minPts.

### 3.2. Cluster Selection from the Condensed Hierarchy

Campello et al. [18] introduced the generic “Framework for Optimal Selection of Clusters” (FOSC) for selecting a flat set of non-overlapping clusters from HDBSCAN’s condensed cluster tree. According to FOSC, it must be possible to compute the specific cluster selection measure for each cluster independently of other selected clusters, i.e., locally. As a second requirement, the measure must be additive, i.e., it must be possible and meaningful to add up the computed cluster values.

Consequently, an optimization problem can be formulated that maximizes the sum of values and also ensures that not more than one cluster is extracted from each branch. This problem can then be solved by traversing the hierarchy tree bottom-up starting from the leaves, deciding for each candidate cluster whether it should be part of the final solution.

#### 3.2.1. Unsupervised Cluster Selection

HDBSCAN’s default selection method eom (excess of mass) is an unsupervised FOSC-compliant cluster selection method and recommended by Campello et al. [4] as the optimal global solution to the problem of finding clusters with the highest stability, where stability is defined as

(1) $$\begin{array}{cc} S(C_{i}) & =\sum_{x_{j}\in C_{i}}\left(\lambda_{max}\left(x_{j},C_{i}\right)-\lambda_{min}\left(C_{i}\right)\right)\\ \; & =\sum_{x_{j}\in C_{i}}\left(\frac{1}{\epsilon_{min}\left(x_{j},C_{i}\right)}-\frac{1}{\epsilon_{max}\left(C_{i}\right)}\right) \end{array}$$

and λ as the inverse of distance value ϵ, i.e., $\frac{1}{\epsilon }$. Hence, λ values become larger from root towards leaves, whereas their corresponding ϵ values become smaller. The value $\lambda_{min}\left(C_{i}\right)$ is the density level at which cluster $C_{i}$ first appears. Subtracting it from the value beyond which point $x_{j}\in C_{i}$ no longer belongs to $C_{i}$ results in a measure of lifetime for $x_{j}$. The sum of all lifetimes within $C_{i}$ leads to the overall cluster lifetime $S(C_{i})$, which is called stability because the clusters with longest lifetimes are considered to be the most stable ones.

The optimization problem for maximizing the sum of cluster stabilities is defined as

(2) $$\begin{array}{cc} \; & \max_{\delta_{2},\ldots ,\delta_{k}}J=\sum_{i=2}^{k}\delta_{i}S\left(C_{i}\right)\\ \; & \mathrm{subject}\;\mathrm{to}\;\left\{\begin{array}{c} \delta_{i}\in \left\{0,1\right\},i=2,\ldots ,k\\ \sum_{j\in I_{h}}\delta_{j}=1,\forall h\in L \end{array}\right. \end{array}$$

with **L** = $\left\{h|C_{h}\mathrm{is}\;\mathrm{leaf}\;\mathrm{cluster}\right\}$ as leaves, $I_{h}$ as the set of clusters on the paths from leaves to the excluded root, and $\delta_{i}$ as Boolean indicator whether the respective cluster is selected or not. Only one cluster can be selected for each branch to avoid overlapping partitions.

To solve this problem, HDBSCAN’s selection algorithm traverses the condensed cluster tree bottom-up and compares the stability value of each node to the sum of stability values of its children. In this way, stabilities are propagated and updated when going up the tree until the cluster with highest stability is found.

#### 3.2.2. Semi-Supervised Cluster Selection

FOSC was introduced not only as framework for unsupervised cluster extraction, but also for semi-supervised selection based on so-called should-link/should-not-link relations [18]. This requires the definition of constraints between pairs of data points prior to the actual clustering. The objective is then to extract clusters from the HDBSCAN hierarchy such that the number of constraint satisfactions over the entire data set is maximized. However, the hierarchy itself is constructed in an unsupervised way without taking constraints into account. This is a major difference to related work in constraint-based hierarchical clustering.

Gertrudes et al. [19] suggested to directly use labels instead of pairwise constraints. The authors argued that this prevents a bias towards should-not-link relations when adding new constraints, and also requires less effort. Hence, they introduced a label-based semi-supervised FOSC procedure where some instances of the data set receive labels that indicate their expected cluster membership. A new selection criterion called $B^{3}F$ measure is used to extract clusters from HDBSCAN’s condensed tree. It can be combined with the unsupervised stability measure to make decisions for instances without pre-labels, or if a more balanced result between stability and prior expectations is preferred.

The $B^{3}F$ measure refers back to the B-CUBED scoring algorithm originally introduced in context of information retrieval tasks and later modified for use as clustering quality criterion [21,22]. In [19], $B^{3}$ Precision of a pre-labeled object $x\in X_{L}$ that belongs to cluster $C_{i}$ is defined as

$$P_{B^{3}}\left(x,C_{i}\right)=\frac{\left|\{x^{\prime }|x^{\prime }\in \left\{C_{i}\cap X_{L}\right\}\wedge class\left(x\right)=class\left(x^{\prime }\right)\}\right|}{\left|\{x^{\prime }|x^{\prime }\in \left\{C_{i}\cap X_{L}\right\}\}\right|}$$

and computes the ratio between the number of pre-labeled instances with the same label as x in cluster $C_{i}$, and the number of pre-labeled instances in $C_{i}$ in total. $B^{3}$ Recall is defined as

$$R_{B^{3}}\left(x,C_{i}\right)=\frac{\left|\{x^{\prime }|x^{\prime }\in \left\{C_{i}\cap X_{L}\right\}\wedge class\left(x\right)=class\left(x^{\prime }\right)\}\right|}{\left|\{x^{\prime }|x^{\prime }\in X_{L}\wedge class\left(x\right)=class\left(x^{\prime }\right)\}\right|}$$

and computes the ratio between the number of pre-labeled instances with the same label as x in cluster $C_{i}$, and the number of pre-labeled instances with this label in total.

The actual $B^{3}F$ measure is the harmonic mean of $B^{3}$ recall and $B^{3}$ precision for the pre-labels instances and thus defined as

$$F_{B^{3}}\left(x,C_{i}\right)=\frac{2P_{B^{3}}\left(x,C_{i}\right)\cdot R_{B^{3}}\left(x,C_{i}\right)}{P_{B^{3}}\left(x,C_{i}\right)+R_{B^{3}}\left(x,C_{i}\right)}$$

Maximizing the overall $B^{3}F$ measure in accordance to FOSC is the objective of the semi-supervised cluster extraction process. Labels provided by the user only serve as preferences, not as hard constraints.

#### 3.2.3. HDBSCAN($\hat{\epsilon }$): Applying a Distance Threshold

When using a low minPts value to cluster data sets with variable densities, it can happen that HDBSCAN’s stability-based method extracts a large number of small clusters in regions with dense data points. Depending on the application, increasing minPts for merging these micro-clusters might not be an option since small clusters in other regions would then be excluded, as demonstrated in our conference paper [8]. To solve the problem of undesired cluster selection on low hierarchy levels, we propose the application of a distance threshold $\hat{\epsilon }$ as additional parameter for HDBSCAN. It makes HDBSCAN act like the fully epsilon-dependent DBSCAN* for data partitions affected by the threshold, and like its typical, epsilon parameter free self in all others.

To formalize the process of cluster selection based on $\hat{\epsilon }$, we use the terms epsilon stable and epsilon stability.

**Definition** **1** (Epsilon stable). *A cluster $C_{i}$ with i={2,...,k} is called epsilon stable if $\epsilon_{max}\left(C_{i}\right)>\hat{\epsilon }$ for a given $\hat{\epsilon }>0$.*

Density level $\lambda_{min}\left(C_{i}\right)=\frac{1}{\epsilon_{max}\left(C_{i}\right)}$ is the level at which cluster $C_{i}$ appears, i.e., separates from its parent cluster. Hence we call a cluster epsilon stable if the split from its parents occurred at a distance above our threshold $\hat{\epsilon }$ (or below the level $\lambda_{min}\left(C_{i}\right)$, respectively). The definition of epsilon stability is given below.

**Definition** **2** (Epsilon Stability).

$$ES\left(C_{i}\right)=\left\{\begin{array}{cc} \lambda_{min}\left(C_{i}\right) & \mathrm{if}\;C_{i}\;\mathrm{is}\;\mathrm{epsilon}\;\mathrm{stable}\\ 0 & \mathrm{otherwise} \end{array}\right.$$

Selecting the cluster with highest epsilon stability on each path of the HDBSCAN condensed hierarchy tree results in the flat set of clusters that must not split up any further w.r.t. $\hat{\epsilon }$ and minPts. Their parents split up at some distance $\epsilon_{min}>\hat{\epsilon }$, which is equal to the $\epsilon_{max}$ value on the level where their children appear. While those nested child clusters are still valid, they are not allowed to split up themselves because either they are leaf clusters or the level $\lambda_{max}=\frac{1}{\epsilon_{min}}$ at which the split would occur is above the threshold. Algorithm 1 shows how to solve the optimization problem of maximizing the sum of ES values in the condensed hierarchy. δ indicates whether a cluster is selected.
**Algorithm 1** Selecting epsilon stable clusters from the hierarchy1.Initialize $\delta_{\left(.\right)}=1$ for all leaves2.Do bottom-up from all leaves (excluding the root):
2.1.If $ES(C_{i})>0$ or $\delta_{C_{i}}=0$, continue to next leaf2.2.Else if $ES(C_{i})=0$ and $ES(C_{PARENT(i)})>0$, set $\delta_{PARENT(i)}=1$ and set $\delta_{\left(.\right)}=0$ for all nodes in $C_{PARENT(i)}$’s subtree


HDBSCAN($\hat{\epsilon }$)’s ES measure is local in the sense that for each cluster, we can decide whether to set the value to 0 or to $\lambda_{min}$ independently of the values of other clusters. It is also additive and does not require any modifications to the construction of the hierarchy. Therefore, it is FOSC-compliant and can easily be added to existing HDBSCAN implementations. It is already integrated in HDBSCAN’s Python implementation [23].

As a solely distance-based selection criterion, HDBSCAN($\hat{\epsilon }$) does not need to be used instead of the default stability measure. If no other cluster selection method is specified, it will return HDBSCAN’s leaf clusters for partitions that are not affected by the given $\hat{\epsilon }$ value. Alternatively, we can run it on top of the clusters selected by eom, or any another method, to merge partitions that lie below a certain threshold. By choosing the threshold smaller or larger, extracted flat clusters will either be more like HDBSCAN clusters (i.e., DBSCAN* clusters on varying hierarchy levels), or more like typical DBSCAN* clusters that cannot be extracted from levels beyond epsilon.

As a rule of thumb, $\hat{\epsilon }$ should be set to a rather small distance value below which the extraction of further subclusters is no longer relevant for the application. The parameter is in general less sensitive than the choice of ϵ for DBSCAN(*): While ϵ by default affects all partitions, $\hat{\epsilon }$ only affects partitions on hierarchy levels smaller or equal than $\hat{\epsilon }$.

From the perspective of semi-supervised clustering, $\hat{\epsilon }$ could also be interpreted as a cluster-level must-link constraint for all nested clusters below $\hat{\epsilon }$. As mentioned in Section 2, Davidson & Ravi [15] used the term ϵ constraint for a disjunction of must-link constraints that enforce a distance that is not larger than epsilon. The application of $\hat{\epsilon }$ could thus be viewed as an ϵ constraint for HDBSCAN’s cluster candidates, but without modifying the process of density-based hierarchy construction, since it was designed as a FOSC-compliant HDBSCAN extension.

HDBSCAN($\hat{\epsilon }$)’s typical use case is the correction of micro-cluster selection, and thereby allows to achieve a combination between DBSCAN* and HDBSCAN clusters as long as $\hat{\epsilon }$ is not chosen too large. Yet, it is also possible to apply $\hat{\epsilon }$*before* the selection process of a different method. In this case, the other method might select ancestors of the maximum epsilon stable clusters, which makes them nested child clusters. We then no longer have the combination of DBSCAN* and HDBSCAN as described above, but the approach can be an advantage in the context of constraint-based cluster selection, where it might happen that certain constraints lead to an early “dead-end situation” [16]. When viewing $\hat{\epsilon }$ from the perspective of a constraint that enforces a distance of at least $\hat{\epsilon }$, the selection of an ancestor by another selection method is no constraint violation: clusters with distances lower than $\hat{\epsilon }$ are still no part of the selection.

We will further discuss and demonstrate this approach in our experiments with constraint-based HDBSCAN.

## 4. Semi-Supervised HDBSCAN Approaches for Radar Data

So far, HDBSCAN has rarely been used in the context of clustering automotive radar measurements. Reasons for that include the high runtime complexity of $O(n^{2})$, but also the view that hierarchies based on a constant distance criterion are inflexible to use [1,2,5]. Schumann et al. [2] tested HDBSCAN on radar reflections from road users, including stationary points. They did not change the stability-based extraction method, but tested two different scaling factors in a *x*-*y*-*v*-*t* space, where *v* refers to velocity and *t* to time. Since HDBSCAN assigned too many static points to clusters of moving objects, it was regarded as not suitable for the task. In the following, we will cluster data sets where stationary points have been filtered out, and then focus on a semi-supervised version with constraint-based cluster selection from the hierarchy. Scheiner et al. [12] presented a generic clustering framework for moving road users, which consists of filtering, clustering, and then merging clusters based on extracted information from the initial clusters. We will follow these three general steps, but based on HDBSCAN instead of DBSCAN, where constructing the HDBSCAN hierarchy for a low minimum cluster size is interpreted as step 2 (clustering), and selecting the final set of flat clusters from the hierarchy as step 3 (merging).

### 4.1. Data Preparation

nuScenes [24] is a large data set for autonomous driving that contains measurements from LIDAR, cameras, and five long-range radar sensors placed on the ego-vehicle, which have a 13 Hz capture frequency. The available radar data sets also include ego-vehicle compensated Doppler velocity values and labels like stationary, oncoming, moving (for reflections facing the opposite direction as oncoming), and crossing moving.

In nuScenes, each traffic scene is 20 s long, and in each scene, a sample (or keyframe) refers to a 2 Hz (0.5 s) frame at a particular timestamp. To get a denser point cloud, it is possible to retrieve sensor measurements aggregated from multiple radar sweeps, i.e., with different timestamps. nuScenes also includes a default filtering option for only returning radar reflections that are likely to be valid.

Cartesian *x* and *y* coordinates are provided in meters relative to the ego vehicle. The *x* coordinate represents front and *y* represents left, i.e., a *y* value of -5 refers to a distance of 5 m to the right side of the ego vehicle.

**Figure 1:**
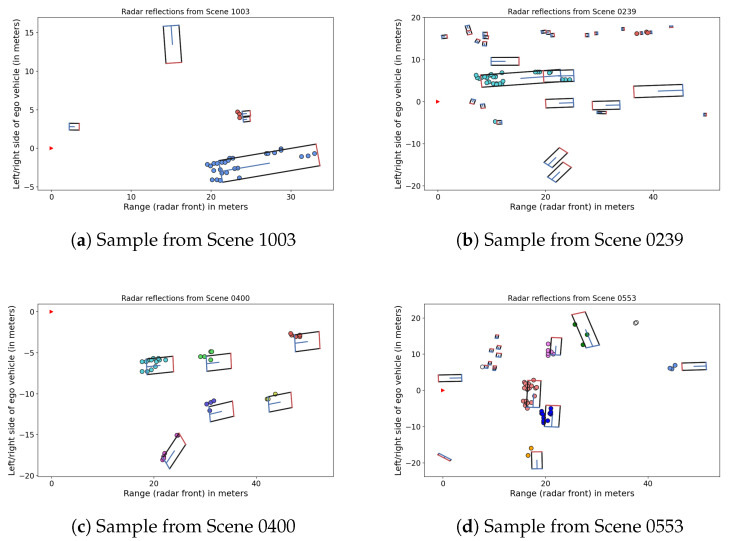
Samples with radar reflections (3 sweeps) and vehicle contours from selected scenes.

Since no ground truth labels for radar measurements were available, we manually labeled the data used for testing HDBSCAN. We selected four exemplary traffic scenes, of which Figure 1 shows typical samples, including their manually assigned labels. Note that it was not the aim of our experiments to cluster a large amount of data, but rather to illustrate the general concept of adjusting HDBSCAN to an application by integrating background knowledge, and to demonstrate that this can improve performance compared to the unsupervised version.

The ego vehicle positions in Figure 1 are marked by red arrows. In some frames, the association of detection points to a moving object was very ambiguous or could not be resolved; these points were labeled as “noise”. Otherwise, only singletons received this label.

We kept nuScenes’ default filtering settings, and additionally filtered out stationary points by only selecting those with the properties oncoming, moving or crossing moving. For simplification and because the differentiation between “oncoming” and its opposite direction “moving” is not always reliable, we marked both oncoming and moving reflections as moving, and all others as crossing moving. For each sample (frame), we retrieved a point cloud of aggregated front radar reflections from 3 sweeps, i.e., a time slot of approximately 0.23 s. We reduced the data to points within 50 m (front) of the ego vehicle coordinate system origin as well as a maximum of 20 m to each side, and removed frames with less than 15 available non-stationary points. For this reason, some scenes contain considerably less frames than others—for instance, only 5 frames from Scene 1003 were clustered, but 32 frames from Scene 0400, as shown in Table 1.

We did not apply any custom metric, but used Euclidean distance in *x*-*y*-*v* dimension in all experiments, where *v* refers to the ego vehicle compensated velocity in meters per second. Further, we used minPts=2 for two reasons: First, some moving objects return only very few reflections, for example pedestrians or distant vehicles. While clustering pedestrians was not our main goal, we wanted to ensure that small groups could still be clustered, like it is the case in Scene 1003 (see Figure 1a). Second, a hierarchy with many small cluster candidates provides more options for selecting the final set of flat clusters than a hierarchy that contains only few large clusters. Nevertheless, it should be noted that choosing such a low minPts is not a requirement for our proposed constraint-based HDBSCAN approach, and depending on data set and application scenario, a higher value might be more appropriate.

### 4.2. Clustering Based on Cluster-Level Constraints

In cluster extraction approaches that are based on either pair-wise constraints or labels, some previous knowledge needs to be available about a fraction of the data set. For instance, if we had clusters from two vehicles parts and could assign a should-link constraint (or simply the same pre-label, as suggested in [19]) to at least two points, one for each part, this information could help to make the right decision in the process of semi-supervised cluster selection.

In our case, information about orientation and velocity of radar points was not enough to automatically retrieve a reliable set of pre-labels prior to the clustering process. We will later use fractions of our manually labeled “true labels” to test label-based HDBSCAN, but first, we introduce our alternative approach, which includes the application of cluster-level constraints. Instead of labeling single instances, aggregated information from cluster candidates within the condensed tree is used to decide whether two sibling clusters should get merged.

Hence, as additional input besides the minPts parameter, we provided background knowledge in form of azimuth/range coordinates, velocities, and moving/crossing moving labels for all data points. Custom threshold values within our constraint specifications (see below) could be included as further parameters; however, since we did not change these settings throughout our experiments, we used hardcoded values instead.

During the process of building the condensed cluster tree, we assigned to each candidate the average value of all of its compensated velocity values. Further, we associated each candidate with the motion property that occurred most frequently within its data points, i.e., either moving or crossing moving. We also noted the provided coordinates within each candidate. The hierarchy itself was not modified in any way.

For our application of clustering moving road users, in particular vehicles, we specified that candidates must not be merged (or in other words, that their parent cluster must not be selected) if one of the following statements is true:

(1) Both clusters face different directions. As mentioned above, we only differentiated between moving and crossing moving.
(2) The difference in average velocity between both clusters is larger than 4 $\frac{m}{s}$.
(3) The distance between their centroids on the car coordinate system’s *x* (front) axis exceeds 15 m. In case of crossing vehicles, the axes are reversed.
(4) The distance between centroids on the *y* axis (left/right side) exceeds 3 m. In case of crossing vehicles, the axes are reversed.

The idea behind using different distance thresholds on front and side axis is that clusters on the same line might belong to the same vehicle, represented by reflections from front and rear. In contrast, clusters that are positioned side by side and facing the same direction are more likely to belong to different vehicles. This assumption is problematic in more complex scenarios such as turning vehicles. However, our main goal was to demonstrate the general concept of using such custom constraints for cluster selection with HDBSCAN. More complex criteria could be developed in the future, including the estimation of object shapes or the integration of information from the environment or other sensors.

Constraints can be computed at each cluster split during HDBSCAN’s condensed hierarchy construction. Alternatively, a search for constraint violations can also be done afterwards in order to retain the option of re-running the selection method with different constraint settings without re-building the hierarchy. In each case, a cluster is added to the collection of constraint clusters if at least one constraint criterion is fulfilled between its two child nodes. Note that leaves cannot be constraint clusters since they have no children.

Flat clusters are selected from the condensed tree by searching on each branch for the highest node that does not violate any constraints and also has no nested clusters with constraint violations. As shown in the pseudo code in Algorithm 2, we first mark all ancestors of constraint clusters as additional constraints in order to ensure that they cannot be selected, even if none of the criteria would be violated when merging their direct children. We can stop the search whenever we reach a node that has already been marked as ancestor constraint before, i.e., while processing a neighbor branch.
**Algorithm 2** Selection based on Cluster-level Constraints**Input:**     Let $T=\{C_{2},...,C_{k}\}$ be the set of cluster candidates of the condensed cluster tree (excluding the root), L⊂T the set of leaf clusters and X⊂T the set of constraint clusters. Let $A_{i}=\left\{j\right|C_{j}$ is ancestor of $C_{i}\}$ denote the set of ancestor indices for a cluster $C_{i}$. Let $\delta_{i}\in \left\{0,1\right\}$ be Boolean indicator whether a cluster is selected or not.    Initialize $\delta_{i}=0$ for i=2,...,k.$\forall i:C_{i}\in X$, go up the tree and set $X=X⋃\{C_{j}\}$ for every unprocessed $j\in A_{i}$$\forall i:C_{i}\in L$, go up the tree and set $\delta_{i}=1$ if $C_{PARENT(C_{i})}\in X$ or root


For every leaf node, we then go bottom up until we reach a constraint node, or the root node if no constraints are available. Its child node $C_{i}$ is selected as cluster.

This bottom-up approach follows the design of other selection methods and leads to a complexity of O(*k*) where *k* refers to the number of cluster candidates in the condensed hierarchy. However, efficiency of the algorithm can be improved by directly accessing and selecting the clusters with lowest indices that are not in the collection of constraint clusters and their ancestors. For example, if two clusters $C_{2}$ and $C_{3}$ are the only (and lowest) constraint nodes, we can directly select their children $C_{4}$, $C_{5}$, $C_{6}$ and $C_{7}$, instead of starting from the leaves. Moreover, if we compute constraints during tree construction, we can already take note of ancestor indices at this stage. HDBSCAN’s overall complexity of $O(n^{2})$ is not increased.

By default, Algorithm 2 does not allow selection of the root node. However, if returning a single cluster should be possible—as it is the case for our data sets, where some frames only contain a single vehicle—we can check whether our selected clusters are the direct children of the root, and whether merging them would violate any constraints. If not, the root cluster can be selected instead of its children.

In the following, we will refer to this selection method based on custom cluster-level constraints as HDBSCAN(constraint). In contrast to HDBSCAN($\hat{\epsilon }$), the algorithm was not designed with focus on FOSC-compatibility, but nevertheless, it can be used in combination with HDBSCAN($\hat{\epsilon }$). Usually, the $\hat{\epsilon }$ threshold is applied after the selection process to merge clusters below a certain threshold, but in our experiments, we applied an $\hat{\epsilon }$ of 1.5 *before* starting Algorithm 2 to avoid that constraints between small clusters at the bottom of the hierarchy lead to an early dead-end in the selection process. In the following, this approach is called HDBSCAN($\hat{\epsilon }$+constraint) or HDBSCAN($\hat{\epsilon }$+c) for short.

### 4.3. Further Clustering Approaches

Regarding the label-based semi-supervised $B^{3}F$ approach—which we will from now on refer to as HDBSCAN(b3f)—it has already been mentioned in Section 3.2.2 that this method guides the cluster selection process, but does not guarantee that two data points with different pre-labels will not be part of the same cluster in the final solution. If we would pre-label all points, the algorithm would return those exact partitions as clusters as long as the hierarchy contains a set of partitions equal to this labeling. If this is not possible with regard to the applied metric (e.g., because the distance between an instance labeled as 1 and an instance labeled as 2 is too small and therefore always part of the same cluster in the condensed cluster tree), the set of partitions closest to the pre-labeled solution would be extracted.

In order to find out how close a flat selection from the constructed condensed tree can get to the ground truth data in case of a hypothetical “perfect” selection method, we included the case of providing 100 percent true labels as pre-labels in our experiments. In addition, we randomly selected 5, 10, and 15 percent of true labels (except points labeled as noise) and used them as pre-labels. These scenarios are meant to simulate cases where a certain fraction of measurements could already be assigned with high certainty based on additional background knowledge. We will refer to them as HDBSCAN(b3f)-5, HDBSCAN(b3f)-10, and HDBSCAN(b3f)-15. Each experiment was repeated 100 times with different seed values; results shown in Section 5 refer to the average scores.

Since some of our clustered frames contain only few data points, we made sure to label a minimum number of two points per frame. Hence, if a frame clustered with HDBSCAN(b3f)-5 had only 20 points, we selected two random pre-labels although this equals a percentage of 10 percent in this data set. As suggested in [18], we used HDBSCAN’s default stability measure to decide ties and to resolve cases where no pre-labels are available.

Besides HDBSCAN(constraint), HDBSCAN($\hat{\epsilon }$+c) and the different HDBSCAN(b3f) versions (including the purely hypothetical approach of providing 100 percent true labels), we included the following methods in our experiments:

- HDBSCAN(constraint) and HDBSCAN($\hat{\epsilon }$+c) with the additional option that for each selected cluster, their child clusters can instead be chosen if this improves the result. We included this approach, in the following called HDBSCAN(alt)—short for alternatives—to find out how often a more accurate solution was only one hierarchy level away from the selected cluster.
- DBSCAN* based on a fixed epsilon value of 4. The threshold value was chosen because it achieved good average results across multiple scenes.
- HDBSCAN(eom), i.e., the original unsupervised HDBSCAN version which selects clusters based on stability.

## 5. Results and Discussion

Table 2 shows clustering results in terms of the commonly used Adjusted Rand Index (ARI), where noise points were treated as singletons. These cannot be viewed as “exact” results: the score for each scene is the average of individual scores for clustered frames within that scene, of which some depend on how certain ambiguities were resolved during the manual labeling process. Besides, it might not always be of actual relevance to the application whether a single point close to a dense point cloud is “correctly” labeled or not, even if it has a negative impact on the score. However, the results do allow a general insight into strengths and weaknesses of different approaches, especially when comparing to the results of HDBSCAN(b3f) with 100 percent pre-labels, which represent the best possible selection of clusters from the given HDBSCAN hierarchy with minPts=2 in *x*-*y*-*v* space.

**Figure 2:**
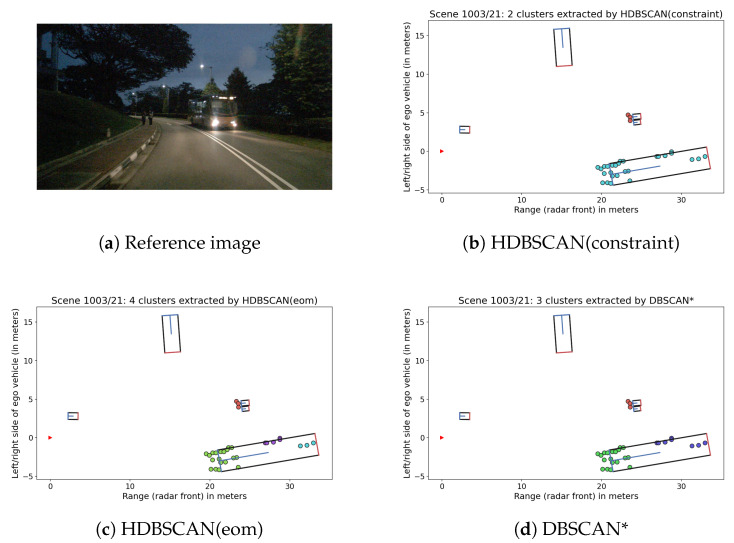
Reference image from nuScenes [24] and clustering results for a sample in Scene 1003.

For scene 1003, with an oncoming bus and pedestrians (see Figure 2a), constraint-based HDBSCAN performs better than unsupervised HDBSCAN and DBSCAN* because it selects the entire bus as a cluster. For DBSCAN*, the fixed epsilon value of 4 is not large enough to include all parts of the bus (Figure 2d), and HDBSCAN(eom)’s stability measure (Figure 2c) also leads to separate clusters. Note that although 1003 is the only night scene out of the four scenes, this does not affect the clustering—as mentioned earlier, radar data is very robust to environmental influences, including lighting conditions. Orientation of the object to the sensor plays a larger role in this case: we have a rather dense point cloud at the front of the vehicle, but only few detection points at the rear and in particular on the side that is facing away from the sensor. This variation in density makes it more difficult to cluster all parts of the bus as one object.

Scene 0239 includes a similar situation, where a large truck is split into two groups of radar reflections at front and rear. As in Scene 1003, unsupervised HDBSCAN has no way of “knowing” that these parts belong together and therefore never selects both of them. The low minPts value also leads to the selection of micro-clusters in some frames, which could be improved by increasing minPts or the application of an $\hat{\epsilon }$ to HDBSCAN(eom). However, this would still not help to merge front and rear parts unless choosing very high values, which is not an option when using the same parameters for all data sets. The same applies to DBSCAN, where a very large epsilon value would be needed to merge both parts, which in turn would cause problems in other situations. In contrast, HDBSCAN(constraint) achieves good results since the lack of constraints between the two vehicle parts allows to select the entire truck.

Scene 0400 represents a very different scenario. It does not include long vehicles, but many cars that are either driving behind each other on the same lane or even switching lanes. Due to the rather compact and dense point clouds per vehicle, unsupervised HDBSCAN shows good performance, almost as good as DBSCAN*. The chosen DBSCAN* threshold works well in this scene, except in some cases where vehicles driving side by side are merged. For HDBSCAN(constraint), lower scores in certain frames are mostly caused by situations where neither of the four specified constraints can prevent that vehicles on the same lane get merged. Note that the same behavior had a positive impact on scores for Scenes 1003 and 0239, where it helped to merge separate parts of the same vehicle. This is because our chosen constraints are too simple to decide the question of whether two separate cluster candidates on the same lane, in relatively close distance and with similar velocity, belong to the same vehicle. Theoretically achievable best results extracted by providing 100 percent true labels as pre-labels indicate that refining our constraints for cluster selection, e.g., by integrating further background information, could lead to better results compared to unsupervised HDBSCAN. This is also reflected by the considerably higher scores for HDBSCAN(alt), which show that the more appropriate choice was mostly just one hierarchy level away.

**Figure 3:**
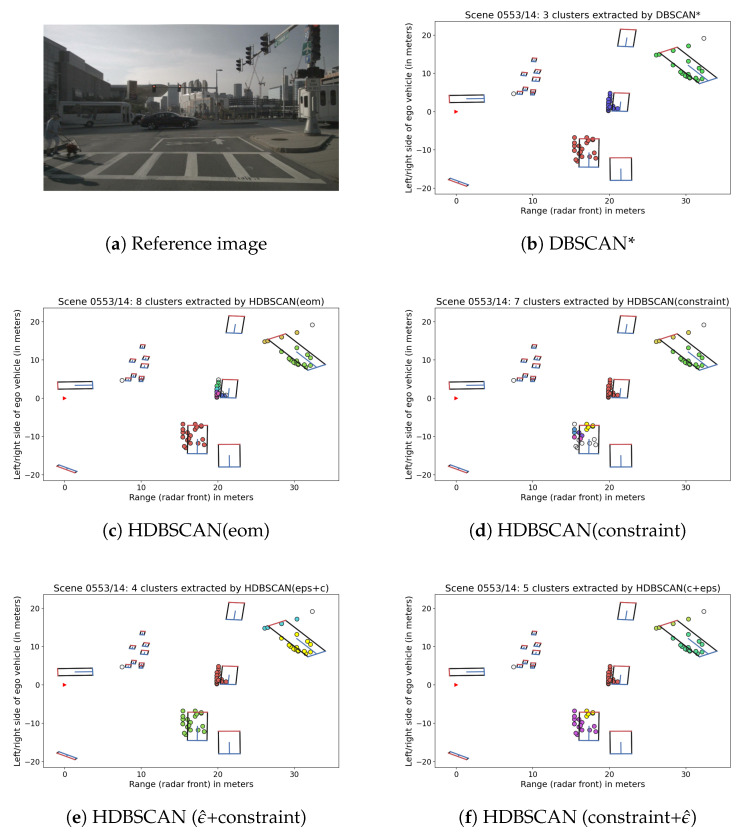
Reference image from nuScenes [24] and clustering results for a sample in Scene 0553.

Scene 0553 (see Figure 3) includes multiple crossing and turning vehicles of different sizes. In some frames, DBSCAN*’s global threshold value merges two vehicles, but in others, such as the frame depicted in Figure 3b, it is well suitable. In the same data set, HDBSCAN(eom) (Figure 3c), selects multiple micro-clusters within the dense point cloud of the vehicle in the middle. This is an issue that happens in particular when using HDBSCAN(eom) in connection with a low minPts value, which results in short cluster lifetimes. The same problem occurs with constraint-based HDBSCAN in a different crossing vehicle, as shown in Figure 3d. Here, the problem derives from the “different directions” constraint. As mentioned before, we differentiated between properties for moving and crossing moving included in the nuScenes data and assigned to each cluster candidate the label that occurred most frequently within its instances. However, these properties are not always reliable, and the label assigned to a cluster is ambiguous if it contains an equal amount of moving and crossing moving instances. In our case, reflections from the respective vehicle were only partly labeled as crossing moving. Because one small cluster at the bottom of the hierarchy was labeled as moving and its sibling as crossing moving, their parent could not be selected and leaf nodes were returned instead. Similarly, another irrelevant different directions constraint can be observed for the rear part of the turning bus in the background.

This is where HDBSCAN($\hat{\epsilon }$) comes into play: as long as we are not interested in clusters within such a small radius, we can apply a low $\hat{\epsilon }$ threshold to merge them. Figure 3e demonstrates that the chosen threshold of $\hat{\epsilon }=1.5$ helped to include all parts of the crossing vehicle by merging micro-clusters before starting the search for constraints. For reference, Figure 3f shows the result for HDBSCAN($\hat{\epsilon }$)’ typical application, where the threshold is used after selecting clusters with HDBSCAN(constraint). It can be seen that $\hat{\epsilon }$ alone is not enough to merge the two remaining parts of the vehicle. Clusters selected by HDBSCAN(constraint) are left unchanged for partitions larger than $\hat{\epsilon }$, which means that the early mistake made by HDBSCAN(constraint) cannot be fixed in this case, except by increasing $\hat{\epsilon }$. In contrast, HDBSCAN($\hat{\epsilon }$+c) merges all parts of the vehicle because the constraint search starts after applying $\hat{\epsilon }$ and the two parts above the threshold no longer violate the different directions constraint: at this level of the hierarchy, the larger cluster contains more data points labeled as crossing moving than moving and is therefore considered as having the same orientation than the smaller cluster. A similar situation could occur when the difference in average velocity between two small leaf nodes is larger than it is between parent clusters at higher levels.

In both cases, $\hat{\epsilon }$ is not large enough to merge the rear of the turning bus, which is why results are still not as good as DBSCAN*’s for this frame. However, note that the purpose of HDBSCAN($\hat{\epsilon }$) is not to simulate DBSCAN* (by choosing a large distance threshold), but to fix the problem of irrelevant cluster selections at low hierarchy levels and otherwise let HDBSCAN decide which clusters to select.

While the potentially different effects of applying an $\hat{\epsilon }$ threshold before or after another selection method should be kept in mind when using HDBSCAN($\hat{\epsilon }$), results for both approaches are the same for all other clustered frames in our data sets. For this reason, we did not list them separately in Table 2. In general, undesired micro-cluster effects in HDBSCAN(constraint) only occurred in scene 0553, which is why HDBSCAN($\hat{\epsilon }$+constraint) results are the same as using HDBSCAN(constraint) alone in the other three selected scenes.

Overall, our cluster-level semi-supervised HDBSCAN version shows promising performance despite being based on very simple constraint definitions. While unsupervised HDBSCAN outperforms HDBSCAN(constraint) in Scene 0400, aggregated results of all four scenes show considerably higher scores for our constraint-based selection method. HDBSCAN(alt) and the theoretically achievable best results indicate that refinement of constraints and integration of additional background knowledge has potential for even further improvement.

Finally, Table 3 shows average results of our experiments with label-based semi-supervised HDBSCAN. The more information in form of pre-labels is available, the more improvement can be seen over the unsupervised approach, which is only used to decide ties or in absence of pre-labels. With 15 percent pre-labels, average scores are slightly higher than HDBSCAN(constraint)’s results for Scenes 0400 and 0553, but lower in the two remaining scenes where different vehicle parts need to be merged.

**Figure 4:**
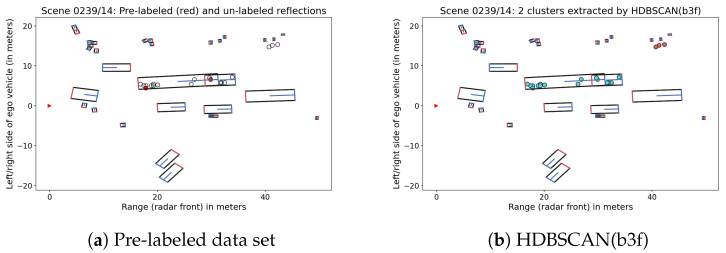
Semi-supervised clustering for a pre-labeled sample in Scene 0239.

Figure 4 demonstrates the possible effect of pre-labels for a frame in Scene 0239. The pedestrian group in the background was clustered in an unsupervised way, but the two pre-labeled data points from the large truck (highlighted in red in Figure 4a) were enough to guide HDBSCAN(b3f) to the selection of the entire truck as one cluster, whereas HDBSCAN(eom) would have chosen separate clusters. On the other hand, the presence of two or even more pre-labels in only one part of the truck would not have improved the result. This illustrates the huge challenge of providing appropriate pre-labels for radar measurements, which is the reason why we instead focused on a cluster-level approach.

## 6. Summary and Outlook

So far, HDBSCAN has rarely been used for clustering radar reflections from moving road users. In this article, we explored whether results achieved by the default unsupervised algorithm could be improved by integrating background knowledge into the process of selecting clusters from the hierarchy. In particular, we designed a semi-supervised HDBSCAN approach that uses cluster-level constraints based on aggregated information from cluster candidates to select clusters from the hierarchy. We combined the task with HDBSCAN($\hat{\epsilon }$), a method that we designed as extension for HDBSCAN to solve the problem of micro-cluster selection at low hierarchy levels when choosing a low minimum cluster size. The method makes use of a distance threshold $\hat{\epsilon }$ as additional parameter. When applying $\hat{\epsilon }$ after selecting clusters of variable densities from the HDBSCAN hierarchy, it can achieve a combination between these HDBSCAN clusters and epsilon-dependent DBSCAN* clusters. As extension to our conference paper [8], we viewed HDBSCAN($\hat{\epsilon }$) from the perspective of constraint-based clustering and pointed out that applying it prior to the cluster-level constraint selection method allows to start the search for constraints from higher levels and thus prevents premature dead-end situations at low hierarchy levels.

We applied our constraint-based selection method to radar data sets from four exemplary traffic scenes, based on the unsupervised construction of HDBSCAN hierarchies with low minimum cluster size. The method showed promising performance despite being based on very simple constraint definitions. In one scene, a micro-cluster effect was observed that derived from the low minimum cluster size and the application of constraints that were not yet relevant at this level of the hierarchy. However, this problem could be resolved by a combination with HDBSCAN($\hat{\epsilon }$). Another limitation was that too many cluster candidates were merged for cars driving in the same direction with similar velocity. To avoid this issue, a refinement of constraint definitions or the integration of additional background knowledge is required, although this can be difficult to achieve in automotive radar data sets where the environment is constantly changing.

We also included label-based semi-supervised HDBSCAN in our experiments. While this version can improve unsupervised results, it depends even more on the availability of accurate background information. For this reason, we found the integration of background knowledge on cluster-level more suitable. However, one advantage of the instance-based semi-supervised HDBSCAN versions is that they are based on soft rather than hard constraints. In the future, our algorithm based on cluster-level constraints should be further developed to also implement soft constraints, e.g., by using different weighting factors for constraint violations. It could also be used to extract multiple alternative solutions for probabilistic multi-target trackers. Moreover, it could be explored whether aggregated cluster information retrieved in one measurement cycle could be used as background information for clustering a subsequent frame, where less reflection points might be available in the same region. Fusion with other sensors to retrieve additional or more accurate background knowledge would be another task for future research. Depending on the application, sensors like LIDAR or RGB-D might be considered [25,26].

In general, the cluster-level approach is not limited to automotive radar data and its typical properties like direction or velocity. Various applications could potentially benefit from the use of aggregated information from HDBSCAN cluster candidates. Future work should explore cluster-level constraints in the context of different types of data, metrics and applications beyond automotive radar data.

## Figures and Tables

**Table 1:** Overview of filtered data sets used for experiments.

Scene	Description	Clustered Frames	Total Points
1003	Oncoming bus and pedestrians	5	128
0239	Long truck, car, pedestrians	17	408
0400	Many cars at highway-like intersection	32	1084
0553	Crossing cars and turning bus	18	756
Total	Data clustered in experiments	72	2476

**Table 2:** Average ARI results for HDBSCAN(constraint) in comparison with other methods. HDBSCAN($\hat{\epsilon }$+constraint) refers to a combined version with HDBSCAN($\hat{\epsilon }$). Both versions were tested with (Alt.) and without (No Alt.) the option of choosing a better alternative from the hierarchy level below. HDB. (eom) refers to default unsupervised HDBSCAN. “Theoretically achievable” refers to the best HDBSCAN cluster selection possible w.r.t. the given hierarchy. All methods are based on Euclidean distance in *x*-*y*-*v* dimension.

Scene	Theoretically Achievable	HDBSCAN(Constraint)		HDBSCAN($\hat{\epsilon }$+Constraint)		HDB. (eom)	DBSCAN*
		**No Alt.**	**Alt.**	**No Alt.**	**Alt.**		
1003	0.97	0.97	0.97	0.97	0.97	0.71	0.74
0239	0.90	0.89	0.90	0.89	0.90	0.48	0.74
0400	0.92	0.82	0.91	0.82	0.91	0.87	0.90
0553	0.96	0.83	0.86	0.88	0.91	0.71	0.83
Average	0.94	0.88	0.91	0.89	0.92	0.69	0.80

**Table 3:** Average ARI results for HDBSCAN(b3f) based on different percentages of pre-labeled instances, with a minimum of two pre-labels. Unsupervised stability was used to decide ties. “Theoretical” refers to the case of 100 percent pre-labels as in Table 2. For all experiments, Euclidean distance in *x*-*y*-*v* dimension was used.

Scene	Theoretical	HDB. (b3f) 5%	HDB. (b3f) 10%	HDB. (b3f) 15%
1003	0.97	0.78	0.81	0.88
0239	0.90	0.66	0.72	0.79
0400	0.92	0.87	0.88	0.89
0553	0.96	0.73	0.80	0.86
Average	0.94	0.76	0.80	0.85

## Data Availability

Publicly available datasets were analyzed in this study. This data can be found here: https://www.nuscenes.org. Code used to analyze the data can be found here: https://github.com/Fusion-Goettingen/Constraint-based-Clustering.
